# Impact of oceanic-scale interactions on the seasonal modulation of ocean dynamics by the atmosphere

**DOI:** 10.1038/ncomms6636

**Published:** 2014-12-15

**Authors:** Hideharu Sasaki, Patrice Klein, Bo Qiu, Yoshikazu Sasai

**Affiliations:** 1 Application Laboratory, JAMSTEC, 3173-25 Showa-machi, Kanazawa-ku, Yokohama, Kanagawa 236-0001, Japan; 2 Laboratoire de Physique des Oceans, IFREMER-CNRS-UBO-IRD, Plouzane 29280, France; 3 Department of Oceanography, University of Hawaii at Manoa, 1000 Pope Road, Honolulu, Hawaii 96822, USA; 4 Research and Development Center for Global Change, JAMSTEC, 3173-25 Showa-machi, Kanazawa-ku, Yokohama, Kanagawa 236-0001, Japan

## Abstract

Ocean eddies (with a size of 100–300 km), ubiquitous in satellite observations, are known to represent about 80% of the total ocean kinetic energy. Recent studies have pointed out the unexpected role of smaller oceanic structures (with 1–50 km scales) in generating and sustaining these eddies. The interpretation proposed so far invokes the internal instability resulting from the large-scale interaction between upper and interior oceanic layers. Here we show, using a new high-resolution simulation of the realistic North Pacific Ocean, that ocean eddies are instead sustained by a different process that involves small-scale mixed-layer instabilities set up by large-scale atmospheric forcing in winter. This leads to a seasonal evolution of the eddy kinetic energy in a very large part of this ocean, with an amplitude varying by a factor almost equal to 2. Perspectives in terms of the impacts on climate dynamics and future satellite observational systems are briefly discussed.

## Abstract (web summary)

Oceanic mesoscale eddies are thought to derive from internal ocean instability. Here, the authors present a high-resolution simulation of the North Pacific Ocean and show that mesoscale eddies are instead sustained by small-scale mixed-layer instabilities triggered by atmospheric forcing in winter.

## Graphical abstract

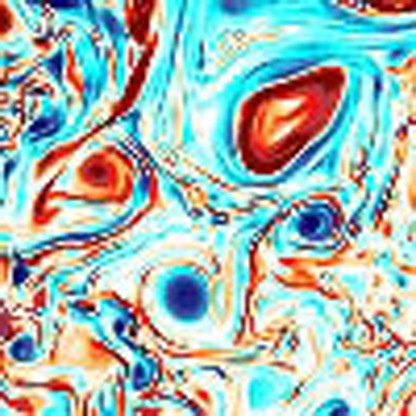

The oceanic flow is known to be highly turbulent involving a very broad range of scales from O(1,000 km) down to O(1 m) and even smaller scales^1^. As such, it is driven by nonlinear scale interactions that can transfer energy upscale or downscale. Oceanic eddies (100–300 km), also called mesoscale eddies, are known to explain not only most of the total kinetic energy (KE) in the oceans^1^, but also most of the turbulent dispersion and transport of tracers such as heat and carbon dioxide in oceanic basins^1^,^2^,^3^,^4^. KE associated with mesoscale eddies is monitored from space by satellite altimeters and these eddies are also explicitly resolved in present-day realistic ocean numerical simulations. Until recently, the broadly accepted paradigm is that these eddies are generated and sustained by the instability of the large-scale vertical current shear (or horizontal gradient of quasigeostrophic potential vorticity (QGPV)) in the ocean interior. Such instability occurs when the sign of QGPV horizontal gradients changes with depth and related studies showed that this depth is around 800–1,000 m. The resulting mesoscale turbulent dynamics is referred to as the Phillips regime^5^ (after the seminal work of Phillips^6^ related to baroclinic instability in the ocean interior) and leads to a velocity spectrum with a *k*^−3^ slope, with *k* being the wavenumber. In this regime, smaller-scale structures (O(1–50 km)), also called submesoscales, are therefore very weakly energetic and have little impact on mesoscale eddies, except in terms of KE dissipation.

However, recent reanalysis of satellite altimeter and *in situ* data^7^,^8^,^9^,^10^ have pointed out that submesoscales are much more energetic than expected in many regions of the oceans, involving a *k*^−2^ spectrum slope over a large range of scales, which suggests an impact of these small scales on larger scales. This questioned the paradigm related to the Phillips regime. Idealized modelling studies of the last 8 years^2^,^11^,^12^,^13^,^14^,^15^ led to the proposition of an alternative interpretation. This interpretation invokes the instability derived from the interaction between the large-scale positive QGPV gradient in the upper layers (induced by large-scale surface density anomalies) and the negative QGPV gradient in the interior. The resulting mesoscale turbulent regime is known as the Charney-like regime^5^,^15^ (after the original work of Charney^16^). Properties of this surface-intensified regime involve a velocity spectrum with a *k*^−2^ slope (instead of a *k*^−3^ slope for the Phillips regime). In this Charney regime, submesoscale structures, which result from frontogenesis triggered by the convergences induced by mesoscale eddies^12^,^15^,^17^, are associated with a vigorous vertical velocity field, indicating a significant transformation of potential energy (PE) into KE at these scales^11^,^14^. The resulting submesoscale KE then feeds up mesoscale eddies and larger scales through an inverse KE cascade^11^,^14^ (that is, KE fluxing from small to larger scales through the nonlinear interactions). However, subsequent investigations of these different regimes in the global ocean^5^ have revealed a dominance of the Phillips regime in energetic eastward currents such as the Gulf Stream and the Kuroshio, whereas the Charney regime may dominate in some other parts of the oceans. This questions the validity of the interpretation of observational results in terms of the Charney-like regime in many regions including the energetic eastward currents.

In the present study, we address this question using a new realistic simulation of the North Pacific Ocean at high resolution (in both the horizontal and vertical directions, see Methods section)^18^ forced by atmospheric reanalysis data. Results highlight the dominance of a class of submesoscales different from what is expected from the Charney regime. These submesoscales are mostly energetic in winter and are related to mixed-layer instabilities^19^ (MLIs, a specific frontal instability occurring when the mixed-layer depth (MLD) is large and vertical mixing vigorous^20^). They emerge in a very large region of the North Pacific Ocean that covers areas such as the Kuroshio and the whole subtropical gyre. These MLIs have been recently revisited in the oceanic case^20^,^21^,^22^,^23^,^24^ but their specific impacts on mesoscale eddies and larger scales are still unknown. We estimate these impacts through a 2-year analysis of the new realistic simulation. Results indicate that the impact of the winter MLIs on mesoscale KE clearly overcomes other dynamical regimes: the resulting submesoscale KE feeds larger scales through an efficient inverse KE cascade, leading to a strong seasonal modulation of the eddy KE (in the 100–300 km band).

## Results

### Seasonality of submesoscales in the North Pacific Ocean

**Figure 1:**
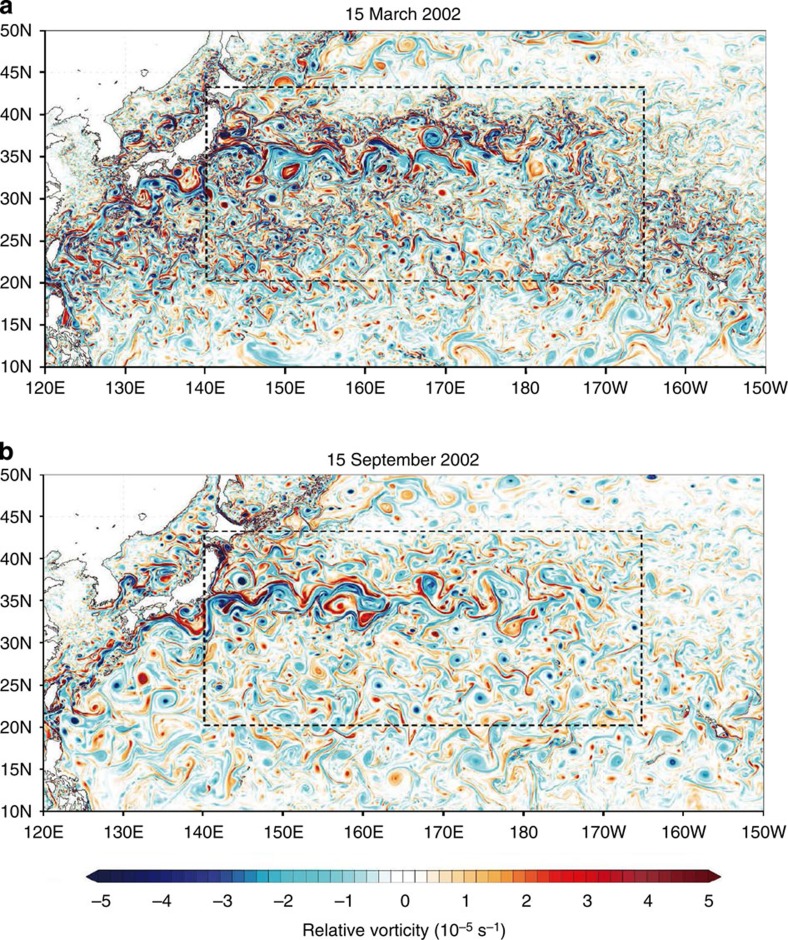
Surface relative vorticity in the Northwestern Pacific. (**a**) On 15 March (**b**) and on 15 September in 2002. The domain (140°E–165°W, 20–43°N) is analysed in the present study.

**Figure 2:**
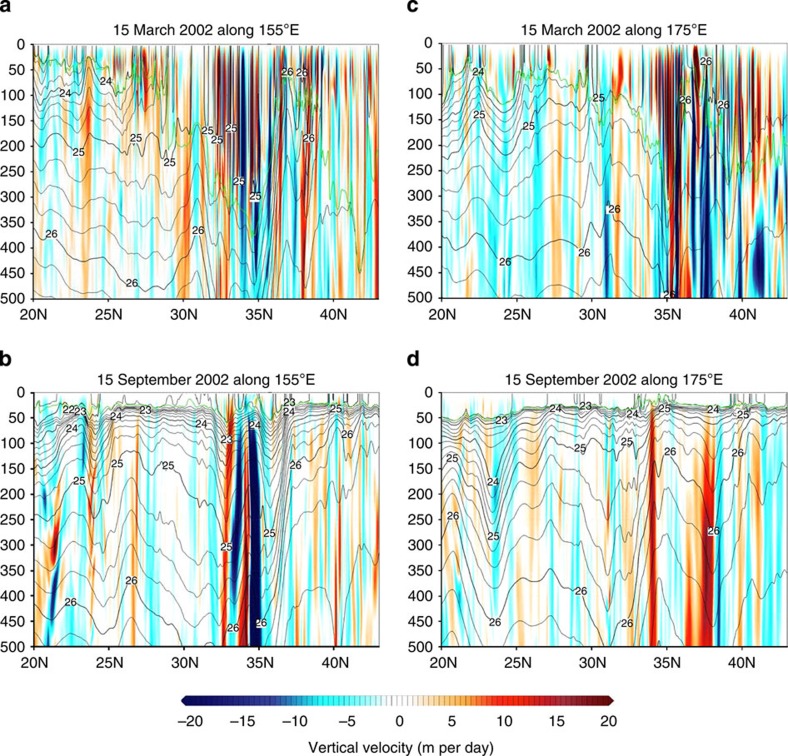
Meridional section of vertical velocity in the Northwestern Pacific. Meridional section along 155°E (**a**,**b**) and 175°E (**c**,**d**) of vertical velocity (colour, m per day), potential density (black contour, σ_θ_) and MLD (green line). (**a**,**c**) On 15 March 2002 and (**b**,**d**) on 15 September 2002. Contour intervals are 0.25 σ_θ_. Depth (vertical axis) is in meters.

**Figure 3:**
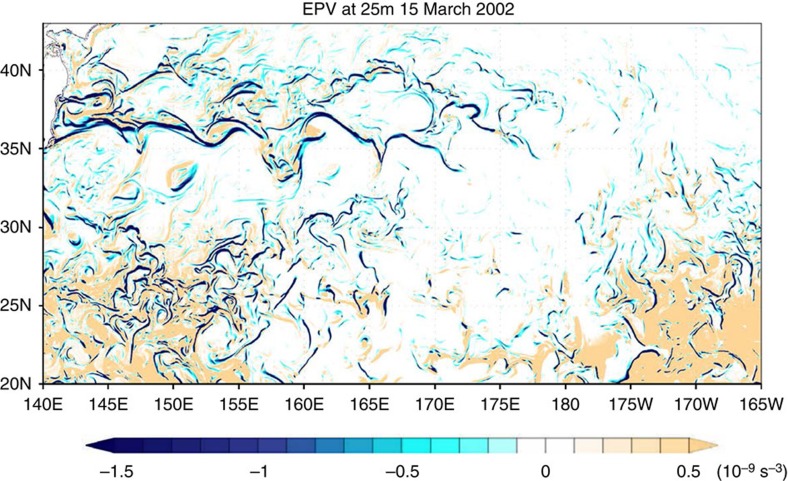
EPV at 25 m depth in the analyzed domain on 15 March in 2002. Negative EPV is found in regions with strong relative vorticity (Fig. 1a) but is principally explained by the vertical shear of horizontal motions (see Methods section).

The surface relative vorticity fields in the western part of the North Pacific Ocean (Fig. 1) reveal a strong seasonality of submesoscales, with winter being much more energetic than summer. This western part captures most of the seasonality of submesoscales of the whole North Pacific Ocean (Supplementary Fig. 1a,b). The field on 15 March 2002 (Fig. 1a) displays mesoscale eddies and a very large number of submesoscale features, such as elongated filaments and small-scale vortices. It is characterized by a strong positive skewness indicating a dominance of cyclones with maximum values close to 2.2 *f* (with *f* the Coriolis frequency). During this period, vertical motions with small scales and large amplitudes (>10 m per day) are present within the ML (Fig. 2a,c), whose depth, averaged over 1 month, varies between 100 and 300 m (Supplementary Fig. 2a). Some vertical motions notably extend well below the ML (Fig. 2a,c). On 15 September 2002 (Fig. 1b), there is a less dense population of submesoscales and the root mean squared (r.m.s.) value of the relative vorticity is smaller by a factor close to 1.7. Amplitudes of vertical motion within the ML, whose thickness is less than 60 m (Supplementary Fig. 2b), are commonly weaker (mostly <5 m per day; Fig. 2b,d). Below the ML, vertical motions have amplitudes larger than 5 m per day, but their horizontal scales are not as small as in winter. The vigorous submesoscale activity in winter is further illustrated by the Ertel potential vorticity (EPV; see Methods section) field at 25 m depth in March (Fig. 3) and in particular by the existence of strong negative EPV (lesser than −2e−9 s^−3^) associated with submesoscale fronts. Such features, not observed in summer, indicate that submesoscale filaments can be affected by frontal/ML instabilities and produce small-scale vortices^20^.

**Figure 4:**
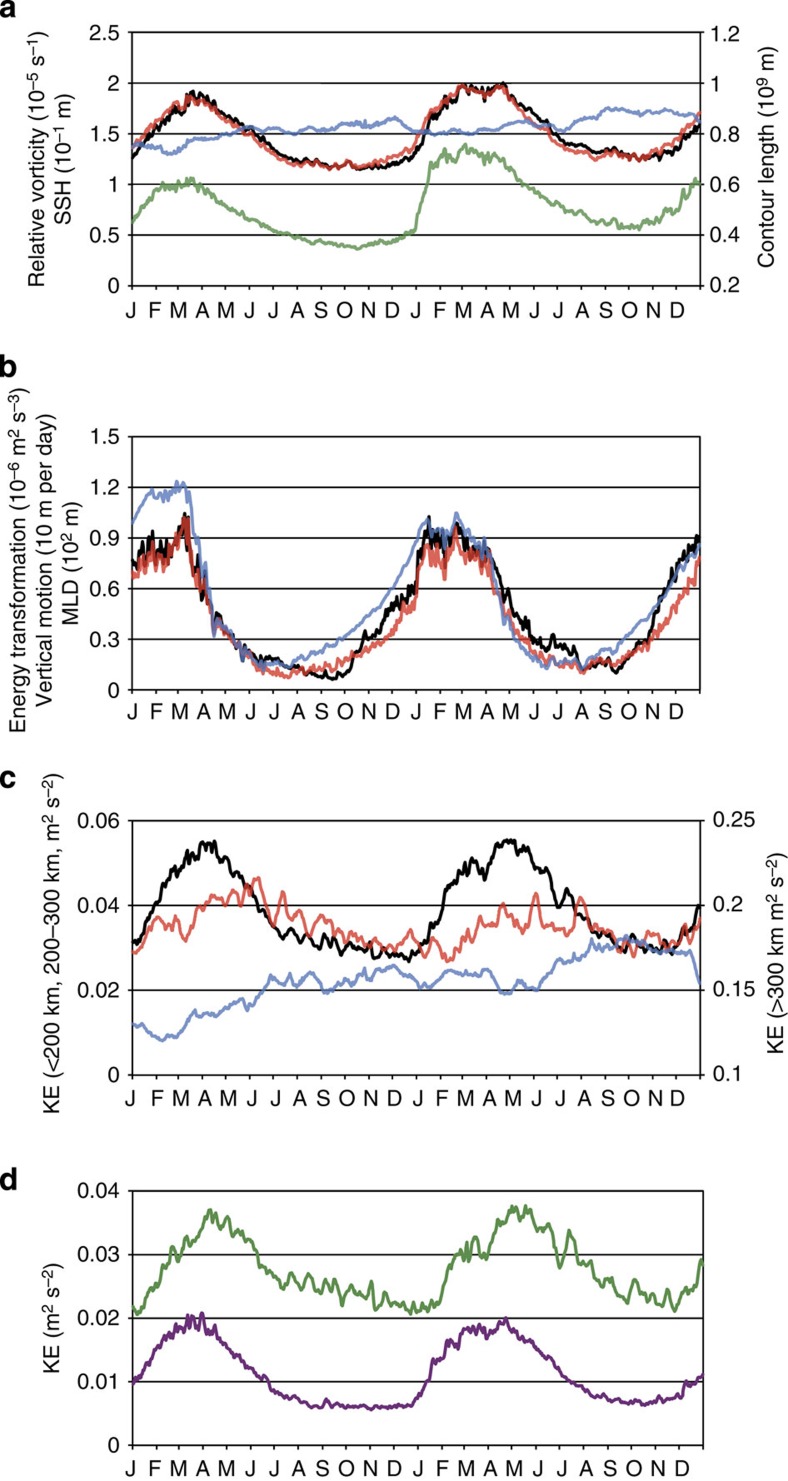
Time series from 2001 to 2002. (**a**) Relative vorticity r.m.s. deduced from velocity at surface (black) and from SSH (red). Contour line length with zero relative vorticity (green) and SSH r.m.s. (blue). (**b**) Transformation from APE to EKE (black), vertical velocity r.m.s. within the mixed layer (red) and mean MLD (blue). (**c**) KE in the 10–200 km band (black), in the 200–300 km band (red) and for scales >300 km (blue). KE for scales larger than 300 km is mostly associated with the Kuroshio jet. (**d**) Partition of the KE in the 10–200 km band: KE in the 10–100 km band (purple) and in the 100–200 km band (green). The analysed domain is 140°E–165°W and 20°N–43°N for **a**,**b** and is 148°E–168°W and 20°N–43°N for **c**,**d**.

These differences between winter and summer are quantified by the time series of the r.m.s. values of relative vorticity estimated from surface motions (black curve in Fig. 4a) in the domain (~4,000 km × 2,300 km) indicated in Fig. 1. This time series exhibits a significant seasonality, with large (small) values in late winter (summer), with a factor close to 1.7 consistent with Fig. 1a,b. Such seasonality is corroborated by the time evolution of the total length of zero relative vorticity contours (green curve in Fig. 4a). The relative vorticity obtained from surface motions has been found to be conspicuously close to the geostrophic relative vorticity (obtained from geostrophic currents estimated by assuming that the Coriolis forces balance the sea surface height (SSH) horizontal gradients in the momentum equations; red curve in Fig. 4a). Correlation between the two time series is 0.97. This indicates that, despite the large r.m.s. relative vorticity values (close to 0.2 *f* in winter), the surface flow is statistically in geostrophic equilibrium as reported in previous studies^11^. This result is also confirmed by the closeness of velocity spectra deduced from geostrophic currents and surface currents (Supplementary Fig. 3): spectra of ageostrophic currents (the difference between surface and geostrophic currents) exhibit amplitudes 10 (4) times smaller at a wavelength of 50 (25) km. In other words, high-resolution SSH data capture not only mesoscale eddies and larger-scale motions but also most of the submesoscale horizontal motions and their seasonality. Interestingly, the time series of the r.m.s. geostrophic relative vorticity (related to SSH by a Laplacian operator) differs much from that of the r.m.s. SSH (blue curve in Fig. 4a): correlation between the two is −0.44. On one hand, this indicates that geostrophic relative vorticity well characterizes the strong emergence of energetic submesoscales in winter. On the other hand, it reveals that the largest scales (that dominate the r.m.s. SSH) are not affected by seasonality and therefore are driven by other mechanisms as discussed later.

### Dynamical impact of submesoscales on larger scales

Figure 4b shows the time series of the transformation of PE into KE (see Methods section; black curve). Its positive sign indicates a net source for KE over the western part of the North Pacific Ocean. This time series displays strong seasonal variations and is close to that of the r.m.s. values of the vertical velocity within the ML (red curve). Both time series correlate well with that of the MLD averaged over the whole domain (blue curve in Fig. 4b): correlation between MLD and vertical velocity (transformation of PE into KE) is close to 0.96 (0.93), which is consistent with the previous studies^22^,^23^. These time series, and in particular that related to the KE source (black curve in Fig. 4b), display a seasonality in phase with the relative vorticity (black curve in Fig. 4a), suggesting that the submesoscale field is seasonally forced. However, the time series in Fig. 4b exhibit a sudden decay in late winter not observed for the relative vorticity. As a result, correlation of these times series with the relative vorticity is merely 0.69. Thus, abrupt decay of these time series, and in particular that related to the transformation of PE into KE, have no immediate impact on the meso/submesoscale field. Since this field is no more forced after this abrupt decay, this suggests that it evolves, at least in the submesoscale range, as a two-dimensional (2D) turbulent flow in free decay^25^. The MLD seasonal evolution is induced by atmospheric forcing as already pointed out by previous studies^23^,^26^, suggesting that production of submesoscales in winter is controlled by large-scale atmospheric forcing via the mixed-layer deepening. These results, however, do not provide detailed information on the different scales involved in this seasonality.

**Figure 5:**
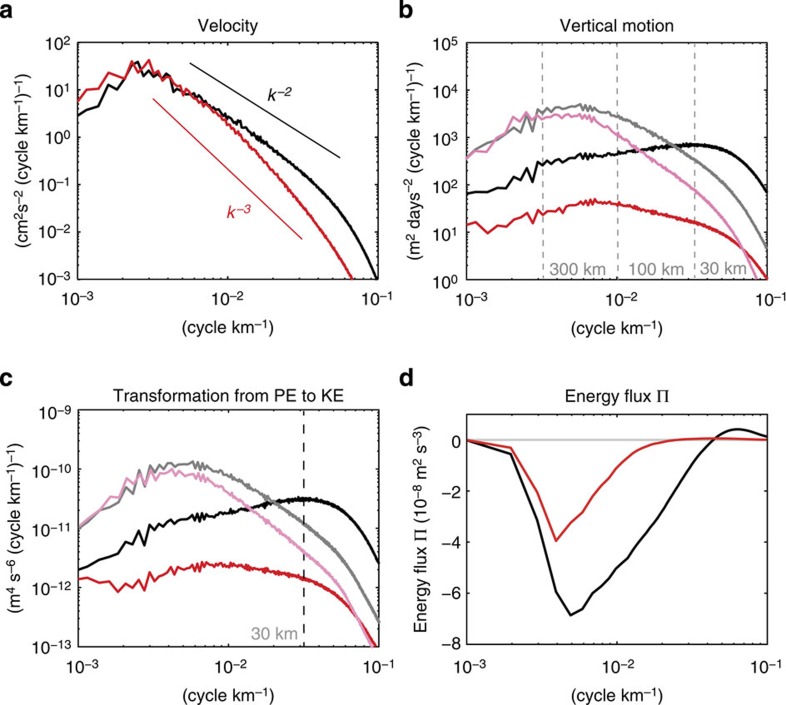
Wavenumber spectra and spectral fluxes. (**a**) Horizontal surface velocity in March (black) and September (red) 2002. (**b**) Vertical velocity averaged in mixed layer in March (black) and September (red) 2002. (**c**) Transformation from PE to KE averaged in mixed layer in winter (February and March; black) versus in summer (from July to September; red). (**b**,**c**) The lines with pastel colours are spectra at the 300 m depth. (**d**) KE spectral fluxes using geostrophic velocities (see Methods section) in winter (black) versus in summer (red). For a given wavenumber, they represent (when positive) the KE that fluxes from smaller wavenumbers to larger ones. The analysed domain is 148°E–168°W and 20°N–43°N for **a**–**c** and is 148°E–166°W and 20°N–43°N for **d**.

To further investigate the production of submesoscales and their dynamical impact (in terms of KE) on larger scales through the nonlinear interactions, several spectral analyses in the wavenumber space have been conducted in four subdomains covering the western part of the North Pacific Ocean and then averaged to get reliable statistics in the full domain (see Methods section). The velocity spectrum in March (black curve in Fig. 5a) displays a peak around 250 km and a *k*^−2^ slope for smaller scales. The September velocity spectrum (red curve in Fig. 5a) exhibits a peak at 300 km with slightly larger amplitude than that in March but involves a steeper slope (close to *k*^−3^) for smaller scales. Differences between these spectral slopes explain the factor 2 increase in winter (relative to summer) displayed by the KE time series in the 10–200 km band (black curve in Fig. 4c). Spectrum of the ML vertical velocity in winter (black curve in Fig. 5b) is closely related to MLIs: it displays a peak at a wavenumber corresponding to a wavelength close to 30 km (that is, close to the most unstable MLI wavelength (24 km), see Methods section), whereas the September spectrum (red curve in Fig. 5b) displays a peak at a wavelength of 150 km.

Spectra of the transformation of PE into KE (Fig. 5c) have been estimated to better highlight the scales involved in the KE source. They display similar features as for the vertical velocity. The winter spectrum (black curve) reveals a peak at a wavelength close to 30 km, whereas the summer spectral peak (red curve) is close to 125 km. Differences between the two spectra highlight the enhanced KE source in winter relative to summer with a dominant KE source at small scale (30 km). Note that these characteristics do not display much dispersion in subdomains (Supplementary Fig. 4).

Impact of submesoscales on larger scales (through nonlinear interactions) has been examined by estimating KE spectral fluxes. From its definition (see Methods section), this quantity, at a given wavenumber *k*, corresponds to the KE that fluxes from smaller wavenumbers (larger scales) to larger wavenumbers (smaller scales). Thus, a positive (negative) value corresponds to a direct (inverse) KE cascade. Results in winter (black curve in Fig. 5d) reveal a significant inverse KE cascade, that is, a KE flux from scales close to 25 km to larger scales (a smaller direct KE cascade is observed for scales smaller than 25 km). As a result, about 70% (40%) of the KE flux at 200 km in winter is explained by the contributions of scales smaller than 100 km (50 km). This highlights the significant dynamical impact of submesoscales on larger scales: a large part of the KE produced at submesoscales feeds up larger scales. During summer (red curve in Fig. 5d), contribution of scales smaller than 100 km (50 km) corresponds to only 25% (0%) of the KE flux at 200 km. Note that these winter and summer fluxes do not display much dispersion in eastern and western subdomains (Supplementary Fig. 5). These spectral fluxes explain the shallower slope of the velocity spectrum in winter (*k*^−2^) relative to summer (close to *k*^−3^) indicating that all scales are fed by winter submesoscales. They also explain the seasonal tendency of the KE time series in the 10–100 and 100–200 km bands (purple and green curves in Fig. 4d): the seasonal variations in the 10–100 km (100–200 km) band display a factor 3 (1.8) increase in winter relative to summer. Furthermore, the KE peaks in the 100–200 km band emerge about 1 month after the ones in the 10–100 km band. This is consistent with the smooth decrease of the relative vorticity (black curve in Fig. 4a) after the abrupt decay of the transformation of PE into KE (black curve in Fig. 4b) mentioned before as well as that of the KE in the 10–100 km band (purple curve in Fig. 4d), whereas the KE in the 100–200 km band continues to increase (green curve in Fig. 4d). Indeed, it takes time for the KE to cascade from small to larger scales. The same tendency is observed for the time series in the 200–300 km band, although the seasonal variations are smaller (factor close to 1.5) and not so pronounced. Furthermore, mean amplitudes of time series in the 100–200 and 200–300 km bands are more than twice than that in the 10–100 km band (Fig. 4c,d). These larger scales are indeed affected by mechanisms other than MLIs (such as the interior baroclinic instability). All these results emphasize, in the western North Pacific Ocean, a significant seasonal modulation of a large part of oceanic mesoscale eddies by large-scale atmospheric forcing (via the winter mixed-layer deepening). This modulation occurs through an energy pathway involving the transformation of PE into KE enhanced at submesoscales and an inverse KE cascade towards larger scales.

The time series of vertical motions below the ML (Supplementary Fig. 6) indicate that the seasonal modulation also affects deeper layers down to 500 m. Spectra of the vertical motions and transformation of PE into KE at 300 m in March and September exhibit different slopes for smaller scales (pastel curves in Fig. 5b,c), suggesting a larger contribution of these scales in winter. However, their spectral peaks (close to 200 km) indicate a dominant KE source at mesoscales, suggesting again instability mechanisms other than MLIs. This is consistent with the KE time series in the 200–300 km band (red curve in Fig. 4c) that exhibits a seasonality not so pronounced as the one for the 100–200 km band. However, the mean amplitude of this time series (200–300 km band) is smaller than that for the 10–200 km band (black curve in Fig. 4c), which emphasizes that most of the KE in the submesoscale–mesoscale band are affected by seasonality induced by the production of submesoscales in winter.

## Discussion

A new realistic simulation of the North Pacific Ocean at high resolution highlights an efficient energy pathway, involving winter frontal instabilities at submesoscale set up by large-scale atmospheric forcings, which catalyses a significant seasonal modulation of the KE over a broad scale range (including submesoscales and mesoscales). This leads, in most of the western part of the North Pacific Ocean, to a factor 2 KE increase in winter relative to summer for this scale range. These results depart from the previous interpretation involving the Charney-like regime about the generation of submesoscales, although the connection with mesoscale eddies (through an inverse KE cascade) and the resulting *k*^−2^ velocity spectrum slope are similar. They much better explain the observational results^7^,^8^,^9^,^10^ in regions known to be dominated by the Phillips-like regime (such as the Kuroshio). At last, they clearly highlight the dominant role of MLIs on the seasonality of the mesoscale eddy KE in a large region of the North Pacific Ocean. An exception is the Subtropical Countercurrent located in the southern boundary of our domain where the seasonality of mesoscale turbulence appears to result from two concurring baroclinic instabilities^27^.

Results of our study point out to the need to further analyse satellite and *in situ* data with a specific focus on the seasonality. The strength of the seasonally varying atmospheric forcing indeed varies from one year to the other and this should affect the interannual variability of the meridional heat fluxes since these fluxes strongly depend on the upper ocean turbulence^2^. The ocean biogeochemical system, on the other hand, is known to strongly depend on the vertical velocity field (that uplifts nutrients from deeper layers into the lighted surface layer) at small scale in the first 300–400 m below the ocean surface^28^,^29^,^30^ as well as on the phase relationship of this vertical velocity field with horizontal motions^30^,^31^. The mechanisms described in this study, which point to the importance of the seasonally varying eddies and small-scale fronts, the ocean weather, may significantly affect this biogeochemical system and consequently (using the same argument as before) affect the part of its interannual variability explained by the atmospheric forcing. At last, monitoring such seasonal modulation requires high-resolution observations on a global scale, which is a major challenge. However, the resulting meso/submesoscale field has been found to be statistically in geostrophic equilibrium at all seasons. This means that such modulation can be diagnosed (using the geostrophic approximation) from SSH data from the future Surface Water Ocean Topography (SWOT)^32^ and Coastal and Ocean Measurement Mission with Precise and Innovative Radar Altimeter (COMPIRA)^33^ wide-swath altimeter missions (see Methods section) that should capture scales 10 times smaller (about 10 km) than what conventional altimeters can do.

Results of this paper highlight the tremendous wealth of the dynamical impact of small scales on the ocean circulation and, as such, suggest further studies with increasing spatial resolution. Thus, the emergence of structures with negative EPV during winter emphasizes the need to further investigate their impact in terms of KE source or dissipation, which requires nonhydrostatic models with higher spatial resolution than the one considered in this study. In the real mixed layer, small-scale turbulence (driven by buoyancy losses and wind stresses) is another important source of energy which also peaks in winter. This turbulence is likely to be confined to scales smaller than the MLIs, but it may interact and modify the instabilities. These interactions are not captured by the model used in this paper because they are too small to be resolved.

## Methods

### Mixed-layer depth

The MLD in this study is defined as the depth at which potential density is different from the sea surface density by 0.03 *σ*_θ_.

### Realistic simulation at high resolution

We analyse output from a high-resolution (1/30° in the horizontal and 100 vertical levels) hindcast simulation of the North Pacific (100°E–70°W and 20°S–66°N) using the Ocean General Circulation Model for the Earth Simulator (OFES)^18^,^34^,^35^. For horizontal mixing of momentum and tracers, a biharmonic operator is applied to reduce numerical noise and the vertical mixing scheme of ref. 36 is used. The model is forced by surface wind stresses and heat fluxes estimated from the 6-hourly Japanese 25-year Reanalysis (JRA-25)^37^. As the initial state, the output on 1 January 2000 from a 1/10° hindcast simulation starting from 1979 is used. The simulation has been integrated for 3 years from 2000 to 2002 (ref. 18) and a longer integration is in progress.

### Ertel potential vorticity

The 3D field of the EPV is estimated by


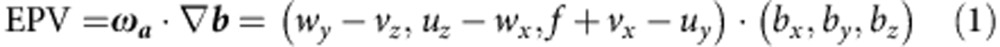


where ***ω***_**a**_ and ***b*** are the absolute vorticity and buoyancy vectors, respectively vectors. A negative EPV should lead to symmetric instabilities^38^.

### Transformation from PE into KE

The net release of PE transferring into KE within the MLD has been estimated from the following equation^20^,^21^,^22^,^23^,


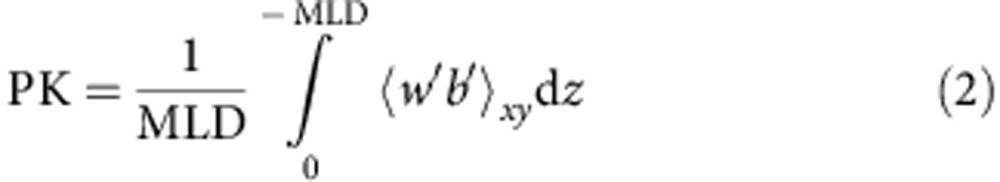


where the angle bracket is averaging over the horizontal box region, *w* is vertical velocity and *b* is buoyancy. The PK in the full domain is estimated by averaging those in the four subdomains.

### Wavenumber spectral analyses

Wavenumber spectral analyses^18^ have been conducted in four subdomains, each with a size of 11° in the longitude and 23° in the latitude. Each subdomain is made double periodic (that is, periodic in both zonal and meridional directions) by doubling its size. To focus on the mesoscale part of the ocean turbulence, zonal mean values are removed. Note that these zonal mean values have been found to affect only scales larger than 300 km. In each subdomain (148–159°E, 159–170°E, 170°E–179°W, or 179–168°W, and 20–43°N), the wavenumber spectra are calculated with the spectral slope estimated down to 10 km. The spectra in the full domain (148°E–168°W and 20–43°N) are then obtained from the average of those estimated in the four subdomains. Only the results in 2002 are shown, because the results in 2001 and 2002 are similar.

### Length scale of the most unstable wave in the mixed layer

The length scale of the most unstable wave in the mixed layer is estimated by using the expression given by the previous studies^20^,^21^,^39^,


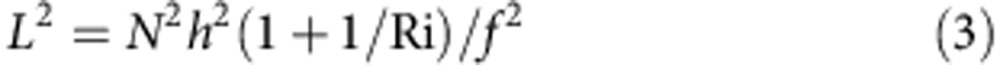


where *N*, *h*, Ri and *f* are respectively the buoyancy frequency, MLD, Richardson number and Coriolis frequency. The resulting wavelength averaged in the full domain in March and September 2002 is 2*πL*=23.8 and 18.0 km, respectively.

### The spectral KE flux

The spectral KE flux is defined as the integral of the local horizontal advective term in the KE equation from the wavenumber *k* to the largest wavenumber *k*_s_ corresponding to the grid size^11^,^40^,


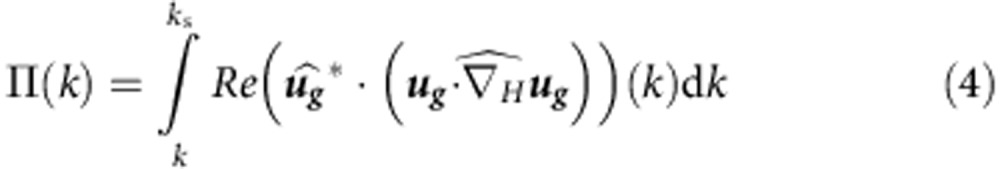


where ***u***_***g***_ denotes the horizontal velocity vector estimated from SSH and ∇_*H*_ is the horizontal gradient operator. 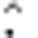 is the horizontal spectral transform and ()* stands for complex conjugate with Re() indicating the real part. To get reliable statistics in the full domain (148°E–166°W and 20–43°N), the spectral KE flux has been estimated by averaging the fluxes calculated in 21 subdomains, 11.5° × 11.5° each, using a Hanning filter. Note that each subdomain is shifted by 5.75° in the both zonal and meridional directions. We use the geostrophic motions, ***u***_***g***_, to estimate the spectral fluxes, although these fluxes are known to be also affected by the ageostrophic motions^11^,^14^. However, this geostrophic estimation is principally used to differentiate the winter and summer regimes.

### SWOT and COMPIRA altimeter missions

Future wide-swath satellite altimeter missions, with a resolution 10 times higher than the conventional nadir-looking altimeters, include the SWOT ( http://swot.jpl.nasa.gov/; http://smsc.cnes.fr/SWOT/)^32^ and the COMPIRA^33^ missions. These new missions should provide an unprecedented global measurement of the SSH, in particular, in the submesoscale range.

## Author contributions

H.S. conceived the central idea and performed the simulation. H.S., P.K. and B.Q. carried out the analysis and wrote the paper. Y.S. provided guidance for the analysis and commented on the manuscript.

## Additional information

**How to cite this article:** Sasaki, H. *et al.* Impact of oceanic-scale interactions on the seasonal modulation of ocean dynamics by the atmosphere. *Nat. Commun.* 5:5636 doi: 10.1038/ncomms6636 (2014).

## Supplementary Material

Supplementary Figures Supplementary Figures 1-6
