# Supplementary material for: Impact of oceanic-scale interactions on the seasonal modulation of ocean dynamics by the atmosphere
Source: Nat Commun. 2014 Dec 15;5:5636. doi: 10.1038/ncomms6636 (PMC4275589; doi:10.1038/ncomms6636)
Supplement: Supplementary Figures — 1-6 [file ncomms6636-s1.pdf]

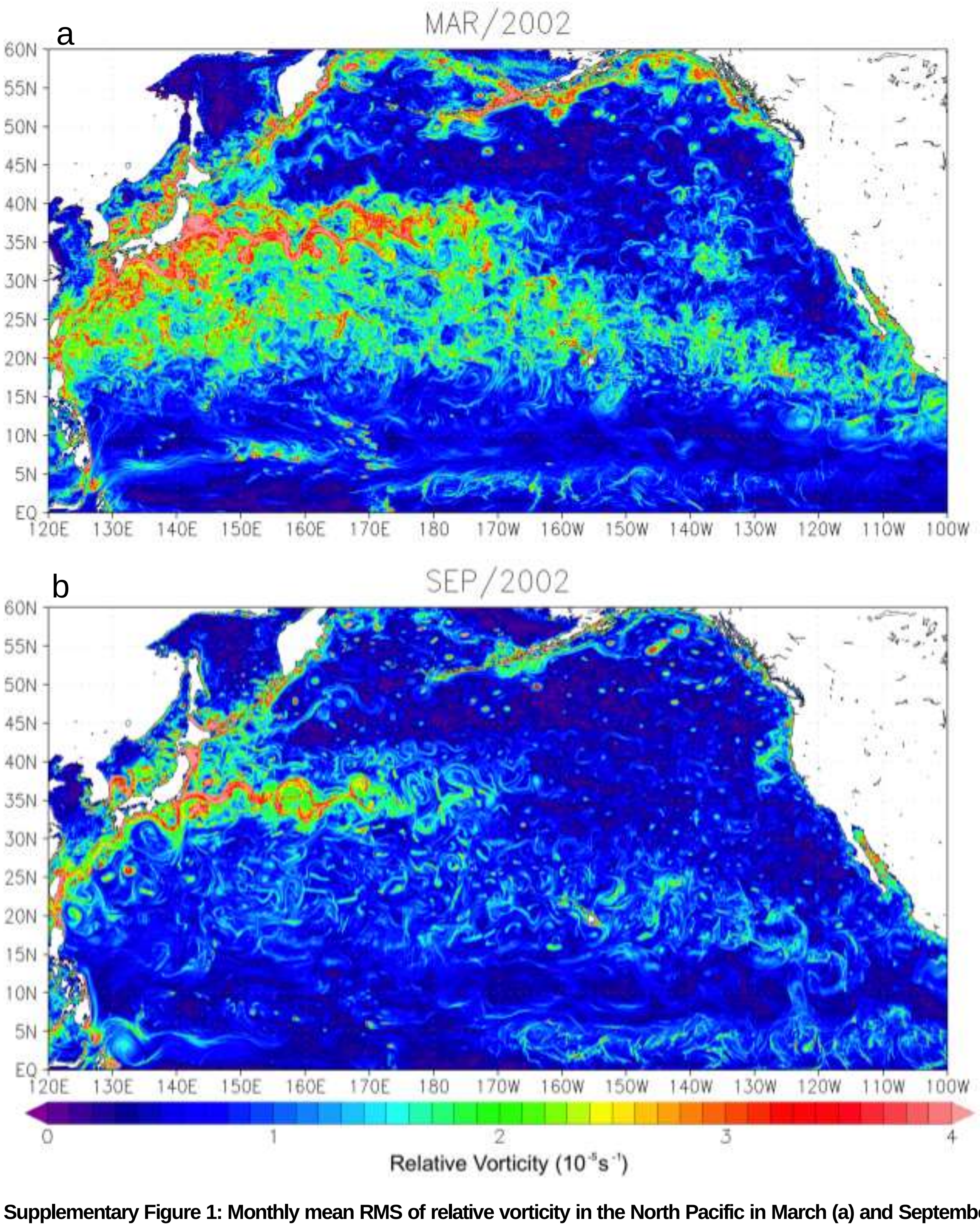

Supplementary Figure 1: Monthly mean RMS of relative vorticity in the North Pacific in March (a) and September (b) of 2002.

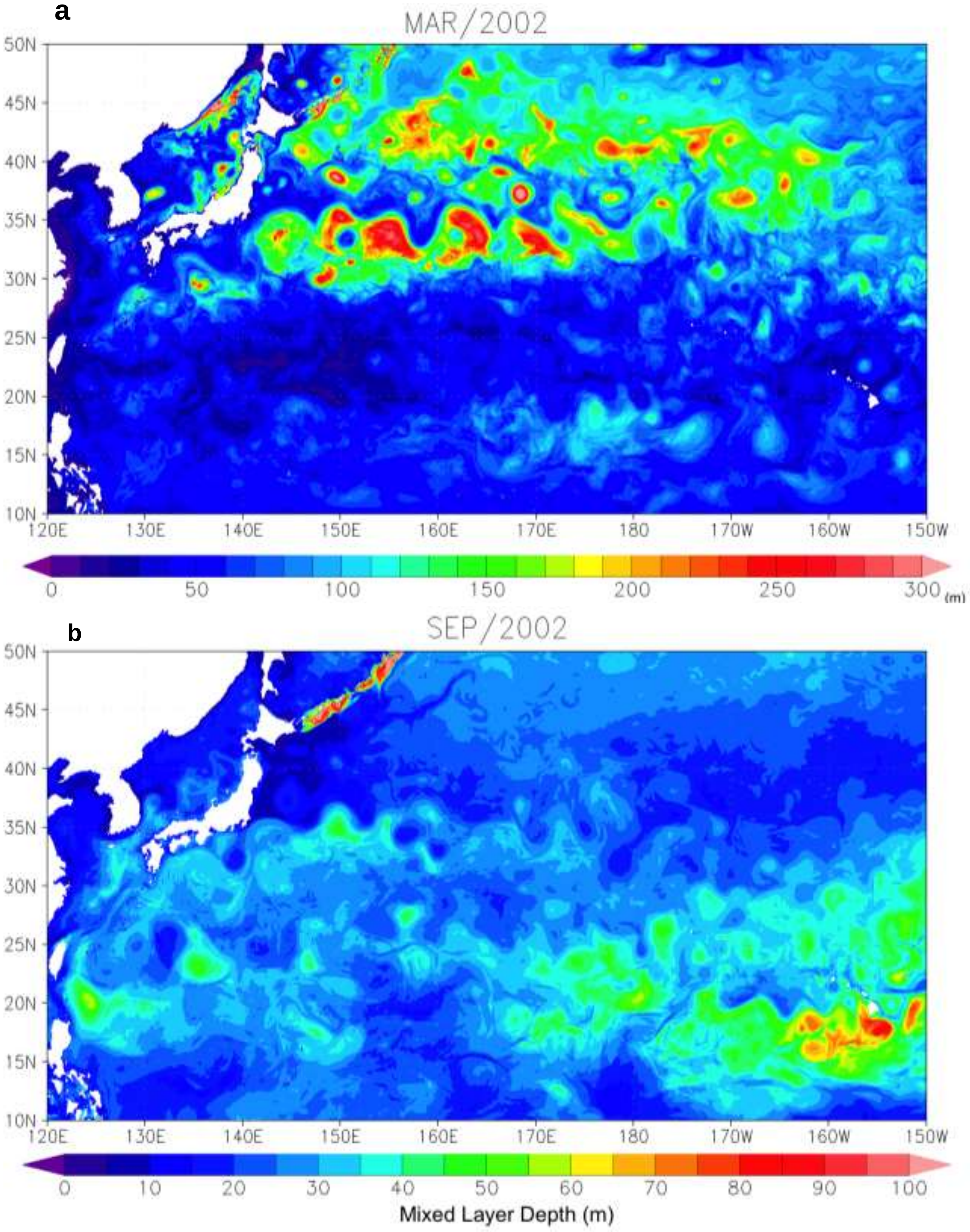

Supplementary Figure 2: Mixed layer depth (m) in (a) March and (b) September of 2002 in the North Pacific.

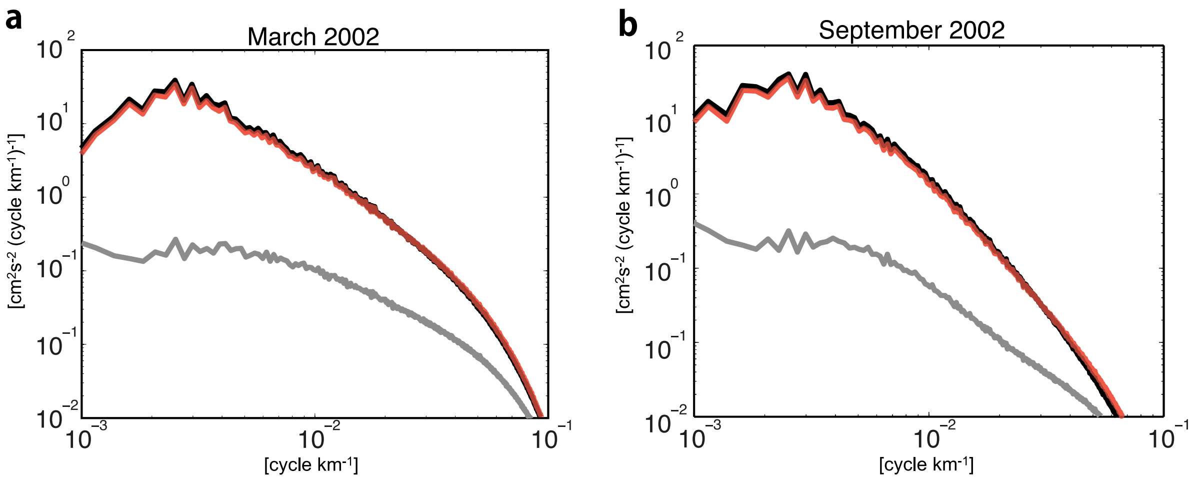

Supplementary Figure 3: Wavenumber spectra of surface velocity (black), geostrophic velocity (red), and ageostrophic velocity (gray) in the box (148°E-168°W, 20-43°N) in (a) March and (b) September of 2002.

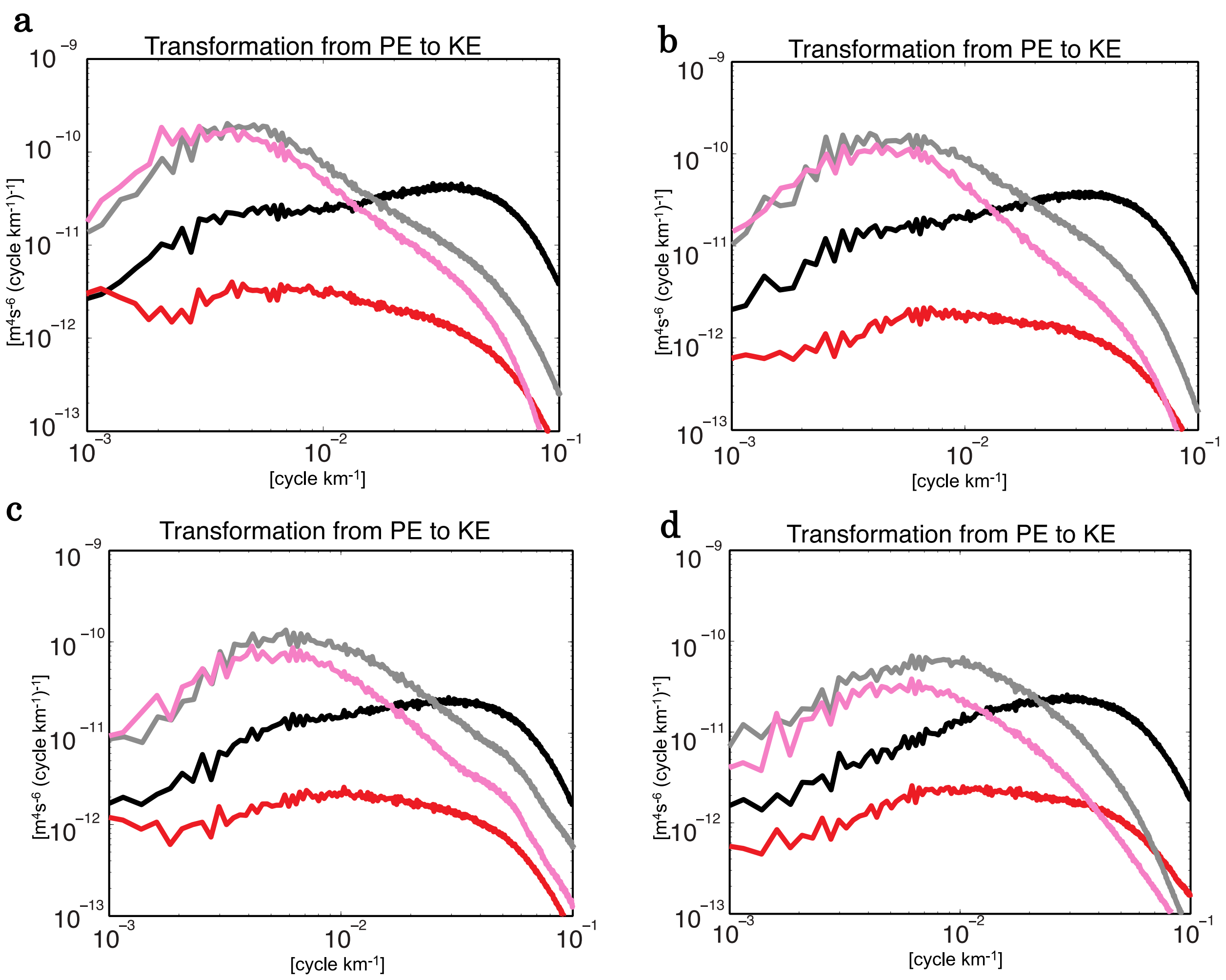

Supplementary Figure 4: Wavenumber spectra of transformation from PE to KE in each subdomain.

Each sub-box is (**a**) 148-159°E (**b**) 159°E-170°E, (**c**) 170°E-179°W, or 179°W-168°W and 20-43°N. Transformation from PE to KE averaged in mixed layer in winter (February and March; black) versus in summer (from July to September; red). The lines with pastel colors are spectra at the 300m depth.

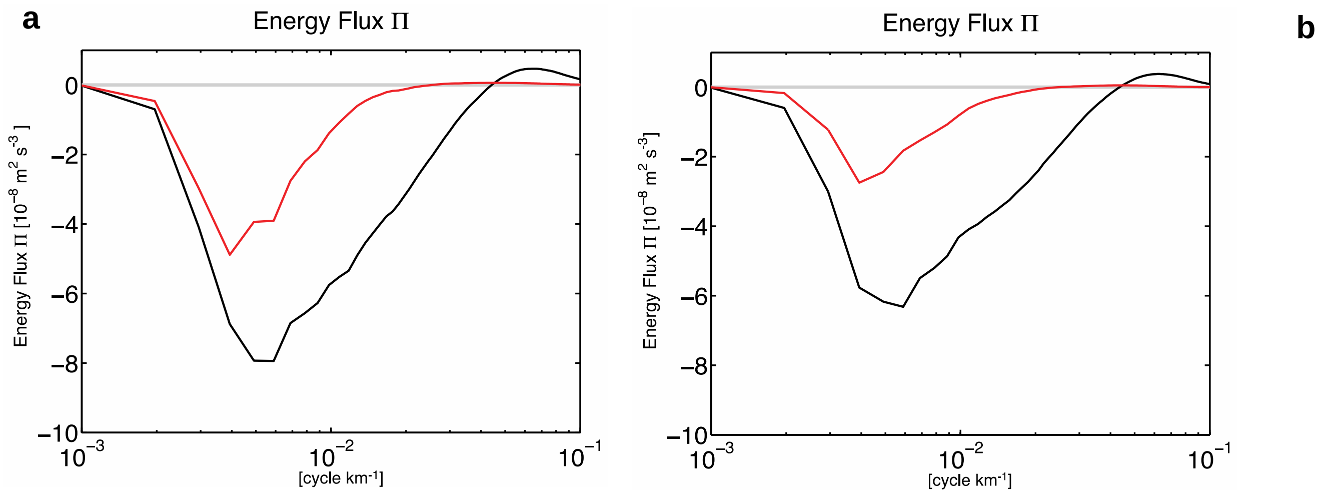

Supplementary Figure 5: KE spectral fluxes in winter (black) versus in summer (red) in the box of (a) 148-171°E or (b) 171°E-166°W and 20-43°N.

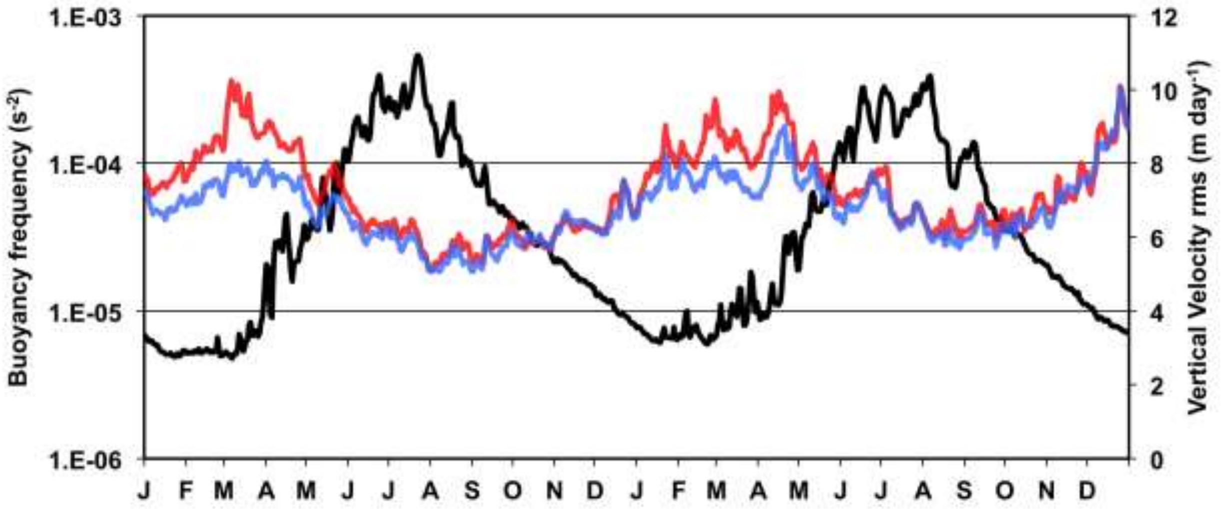

Supplementary Figure 6: Time series of mean buoyant frequency averaged in the mixed layer (black) and vertical velocity rms at 300m (red) and 500m (blue) depth from 2001 to 2002 in the box region of 140°E-165°W, 20-43°N.
